# Early-life experience affects honey bee aggression and resilience to immune challenge

**DOI:** 10.1038/srep15572

**Published:** 2015-10-23

**Authors:** Clare C. Rittschof, Chelsey B. Coombs, Maryann Frazier, Christina M. Grozinger, Gene E. Robinson

**Affiliations:** 1 Carl R. Woese Institute for Genomic Biology, Department of Entomology and Neuroscience Program, University of Illinois Urbana-Champaign, Urbana, IL, 61801; 2 Department of Entomology, Center for Pollinator Research, The Pennsylvania State University, University Park, PA, 16802; 3 Department of Molecular and Cellular Biology, University of Illinois Urbana-Champaign, Urbana, IL, 61801.

## Abstract

Early-life social experiences cause lasting changes in behavior and health for a variety of animals including humans, but it is not well understood how social information ‘‘gets under the skin’’ resulting in these effects. Adult honey bees (*Apis mellifera*) exhibit socially coordinated collective nest defense, providing a model for social modulation of aggressive behavior. Here we report for the first time that a honey bee’s early-life social environment has lasting effects on individual aggression: bees that experienced high-aggression environments during pre-adult stages showed increased aggression when they reached adulthood relative to siblings that experienced low-aggression environments, even though all bees were kept in a common environment during adulthood. Unlike other animals including humans however, high-aggression honey bees were more, rather than less, resilient to immune challenge, assessed as neonicotinoid pesticide susceptibility. Moreover, aggression was negatively correlated with ectoparasitic mite presence. In honey bees, early-life social experience has broad effects, but increased aggression is decoupled from negative health outcomes. Because honey bees and humans share aspects of their physiological response to aggressive social encounters, our findings represent a step towards identifying ways to improve individual resiliency. Pre-adult social experience may be crucial to the health of the ecologically threatened honey bee.

Early-life social interactions can cause brain structural changes^1^ and modifications to the epigenome^2^ resulting in behavioral effects that persist throughout life. This phenomenon has been demonstrated across a range of vertebrate species including humans, but in general, the mechanisms by which social information becomes biologically embedded are poorly understood^3^. In humans, one prevalent example of such early-life social effects occurs in the context of aggression. Exposure to social aggression including domestic abuse, bullying, and neighborhood violence can lead to increased aggression and violence later in life, as well as increased disease risk^3^,^4^,^5^. Understanding the mechanistic links between socially induced variation in behavior and negative health outcomes is important for fostering individual resilience and remediating the consequences of social stress.

Increasingly, studies suggest that some of the molecular mechanisms that transduce aggression-related social information are broadly shared across animal species^6^. For example, a recent comparative study involving honey bees (*Apis mellifera*) showed that the brain transcriptomic response to an aggressive social encounter in adults includes changes in *NF-kappa B* signaling^7^, which is also thought to mediate the negative health outcomes associated with exposure to social stress in humans^8^. Thus honey bees may provide comparative insights into the mechanistic basis of the behavioral and health consequences of exposure to social aggression.

Adult honey bees exhibit collective nest defense against mammalian predators and conspecific robber bees from other colonies. The rapid defensive response is socially coordinated by pheromone cues, but aggression is also socially modulated over longer time scales allowing colonies to adjust their behavior in response to local ecological conditions^9^,^10^,^11^. No study, however, has assessed whether early-life (i.e., pre-adult) social experience also influences adult aggression in the honey bee. Because early-life influences are fundamental to human social response, such an effect in the bee would provide increased support for its use as a comparative system for social aggression. Moreover, because honey bee colony performance is dependent on individual behavioral plasticity during adulthood, the existence of pre-adult social effects could have broad implications for studies of colony social organization, function, and health.

In the current study, we performed cross-fostering experiments to manipulate the early-life social environment for individual honey bees. We then assessed the adult behavioral effects of this treatment using a lab-based aggression assay. Finally, we evaluated whether, like humans, an aggressive early-life social environment has negative health consequences by measuring susceptibility to a health stressor, topical pesticide treatment, and evaluating the relationship between aggression and ectoparasitic mite presence.

## Results and Discussion

**Figure 1:**
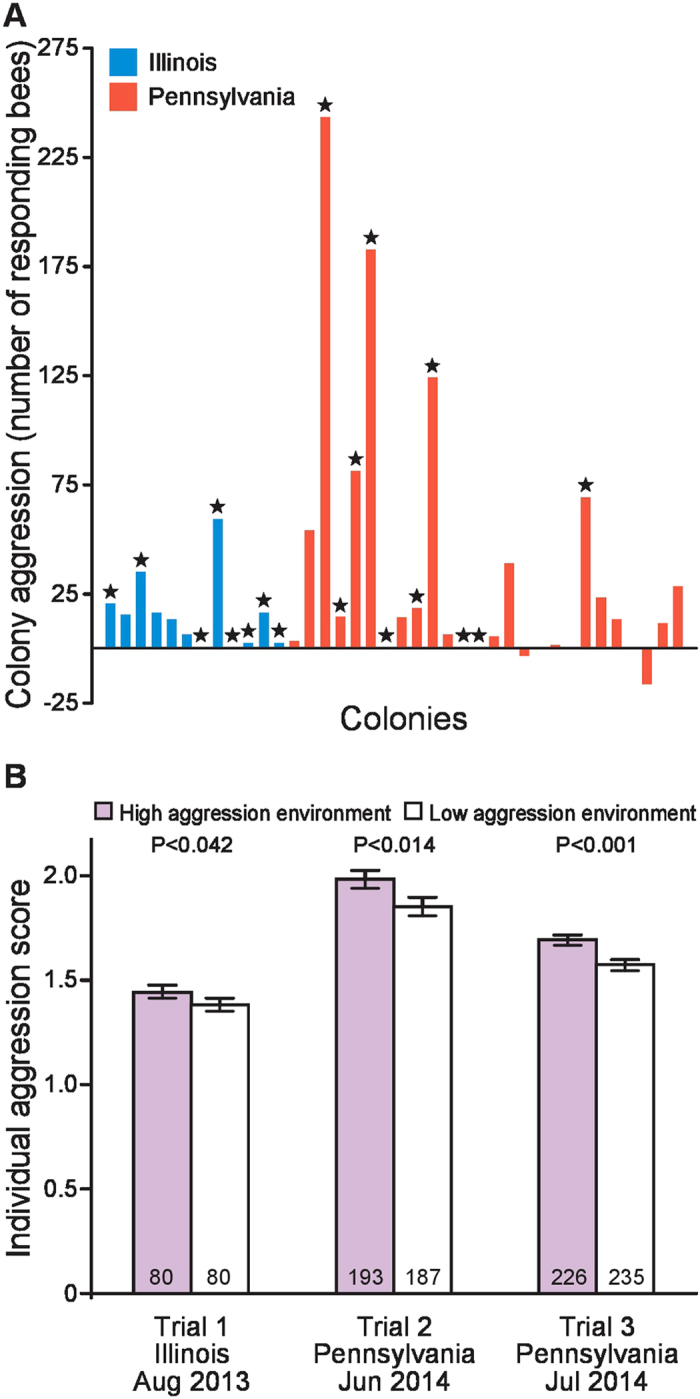
Aggression scores for foster colonies and cross-fostered bees. (**A**) Colony aggression was assessed as the number of individuals responding to a controlled presentation of the primary component of honey bee alarm pheromone. 18 colonies were selected as hosts for the cross-fostering experiment (indicated with stars). (**B**) In three trials spanning two geographic areas, 18 unique genotypes, and 18 unique foster colony environments (N = 48 genotype by foster colony combinations), early-life environment was a significant predictor of individual aggression score (one-tailed *t*-tests). An aggressive early-life environment caused a 10–15% increase in adult aggression, and a pooled analysis across trials yielded similar results (see text). The total number of groups assayed for behavior is denoted in each bar. Log-transformed individual aggression scores (mean +/− S.E.M.) displayed are based on tallies of aggressive behaviors in a laboratory Intruder Assay (see Methods and Supplementary Fig S1).

We manipulated honey bee early-life social experience by fostering juvenile individuals in either high or low aggression colonies. We assayed 38 honey bee colonies in the field for aggression to select foster colonies to host brood (Fig. 1A); those in the top and bottom 20% of the distribution were designated ‘high’ and ‘low’ aggression colonies, respectively. Foster colonies received honeycomb frames containing eggs of various genotypes (laid by queens selected at random from a different set of colonies, see Supplementary Information) and were then left undisturbed until one day prior to adult emergence (~18 days), when we transferred the honeycomb frames to a laboratory environment.

Upon emergence in the laboratory, adults were placed in small groups in cages and maintained under common conditions for 7 days prior to aggression assessment; this ensured our results reflect variation in pre-adult experience only. We measured aggression for 8-day-old bees using an assay that quantifies aggressive behaviors displayed by a group of bees towards a foreign intruder bee^12^,^13^,^14^. For each group, we summed total aggressive behaviors and calculated per-bee aggression scores. In three separate trials, individuals raised in high aggression foster colonies were 10–15% more aggressive compared to individuals raised in low aggression foster colonies (Fig. 1B). A pooled analysis of all data showed a highly significant effect of colony environment on aggression (analysis of z-scores: whole model: F_18,982_ = 2.43, P < 0.0008, foster colony aggression level: F_1_ = 17.3, P < 0.0001, worker genotype: F_17_ = 1.6, P < 0.069; the interaction term was non-significant and so excluded). Furthermore, siblings housed in either high or low aggression foster colonies in the same trial showed a positive relationship between foster colony aggression and individual aggression in 14 of 15 cases (foster colony aggression [genotype]: F_15_ = 1.55, P < 0.08, Supplementary Fig S1).

In contrast to prior studies using field-based aggression assays, the lab-based assay allowed us to measure social effects on aggressive behavior without the confounding social input of the colony environment at the time of assessment^15^,^16^. However, we found that colony level variation in aggression is larger than individual variation (Fig. 1A vs. B), which could reflect differences in behavioral assays, or indicate that social influences during adulthood ameliorate or accentuate early-life effects. The laboratory test reflects the natural context of ejecting a non-nestmate who is attempting to gain colony entry and steal honey. This context, both in the field and in the lab, results in a relatively mild aggressive response compared to the rapid escalation that accompanies the response to a mammalian predator, simulated in the colony-level assay. However, our data showed that behavioral effects were consistent across all three trials, demonstrating a lasting effect of early-life social environment on aggression, generalized across a wide range of honey bee genotypes and foster environments, two different geographical locations, and three time periods distributed across the summer season (Fig. 1B).

Body size is highly sensitive to larval food provisioning in honey bees^17^ and is associated with variation in aggression in many animals^18^. We measured wing length (an established measure of body size^19^, Supplementary Fig S2) of cross-fostered bees from three genotypes to assess the possibility that individuals kept in low aggression foster colonies failed to receive adequate nutrition for appropriate growth. Individual genotype was a significant predictor of size, but there were no size differences as a function of foster colony aggression level (whole model F_5,113_ = 5.77, P < 0.0001, genotype: F_2_ = 12.84, P < 0.0001, foster colony aggression*genotype: F_3_ = 1.11, P < 0.35). These results suggest colony social environment influences aggressive behavior without overt changes in physical development and morphology.

**Figure 2:**
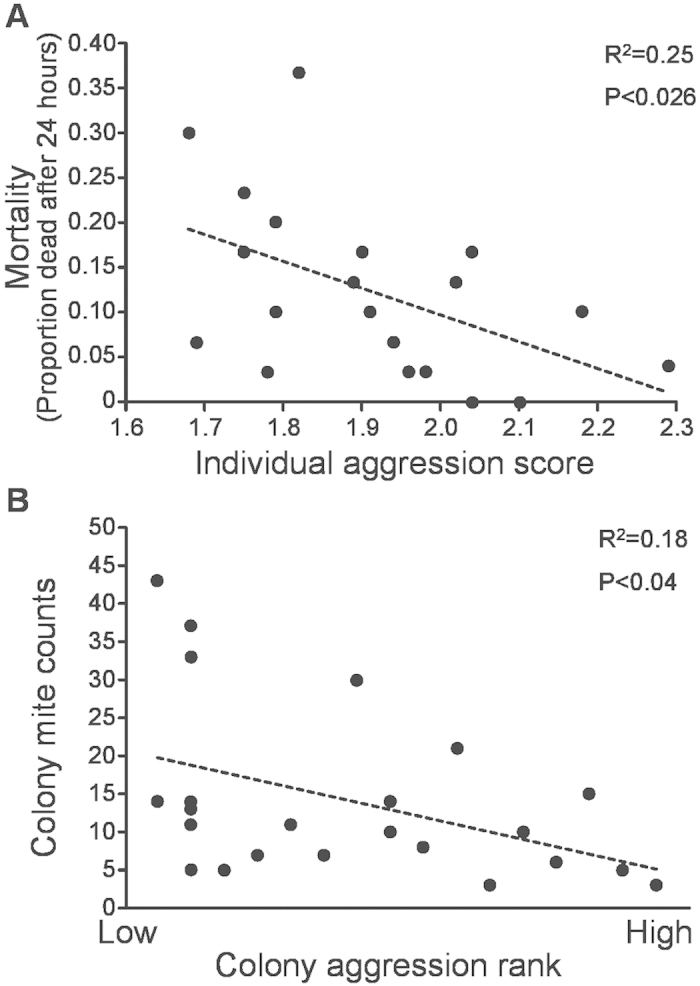
High aggression is associated with resilience to immune challenge. (**A**) Aggression level was significantly negatively correlated with mortality following topical treatment with the neonicotinoid pesticide acetamiprid. Individual aggression score represents a log-transformed mean calculated from all groups tested in the behavioral analysis for each genotype by foster colony combination in Trial 2 (N = 20 combinations). Pesticide susceptibility was assessed using a different set of bees from those in the behavioral experiments. These bees emerged at the same time and were housed and fed identically prior to treatment. (**B**) Mite infestation at the colony level is negatively correlated with colony aggression. Data show a subset of colonies from Fig. 1A (Pennsylvania). Colonies are ranked from low to high aggression level (ties received the same rank).

In humans, behavioral effects of early-life exposure to social aggression are often accompanied by negative health consequences^8^,^20^. We evaluated evidence for such effects in honey bees by assessing whether environmental effects on behavior predicted adult susceptibility to acetamiprid, which belongs to a class of pesticides (the neonicotinoids) known to impose an immune challenge^21^,^22^. We applied acetamiprid topically (1.2 μg/bee, selected as an approximate LD_50_ based on a pilot assessment, see Supplementary Fig S3) to 7-day-old adults from each genotype-by-foster colony combination in Trial 2 of the above experiment (N = 20 combinations, N = 30 bees/combination; these bees were distinct from those used in the behavioral assays). We assessed mortality 24 h after treatment. Aggression level, which is significantly influenced by pre-adult social environment, was negatively correlated with pesticide susceptibility (R^2^ = 0.25, P < 0.026, Fig. 2A). Thus unlike in humans, exposure to an aggressive social environment is associated with positive, not negative, outcomes in response to additional health stressors.

High aggression also was associated with lower parasitization by *Varroa destructor* mites. As in previous studies^23^,^24^, there was a significant negative correlation between colony aggression and mite infestation measured at the colony level (R^2^ = 0.18, P < 0.04, Fig. 2B). Moreover, bees reared in more aggressive foster colonies had lower numbers of mites upon emergence (mites were counted on bees following behavioral tests in Trials 2 and 3, Wilcoxon Tests, Z = 5.48, P < 0.0001; Z = 3.076, P < 0.0021). Variation in adult grooming or hygienic behaviors^23^, or reduced mite reproductive success on pupae^24^ could minimize the proliferation of mites in high aggression colonies. However, our results could also reflect the opposite relationship, i.e., that mite feeding on bees during development negatively influences aggression, and this effect manifests at both the colony and cross-fostered individual levels.

We performed additional analyses that indicate that mite feeding does not explain the effects of the pre-adult environment we observed. First, aggression levels of cross-fostered bees were largely independent of mite presence on emerging bees (which would be indicative of mite feeding during development): in Trial 3, we observed only a weak negative correlation between individual aggression and the number of mites present on bees (R^2^ = 0.03, P < 0.002), and there was no such relationship in Trial 2 despite higher overall mite levels (see Supplementary Information). Moreover, for Trial 3, we found that the influence of the pre-adult environment on aggression was consistent regardless of whether or not mites were present on cross-fostered bees (Supplementary Fig S3). Thus while mite feeding may influence aggression, it does not explain the finding that early-life exposure to an aggressive social environment causes increased aggression later in life.

Mites transmit viruses, and significant feeding can suppress the immune system resulting in a spike in viral titers^25^. We evaluated titers of deformed wing virus (DWV), which is the most common virus transmitted by *Varroa* mites and an indicator of significant mite feeding^25^. We used quantitative PCR to compare DWV titers for cross-fostered bees with high versus low mite numbers for Trial 3. We detected no DWV in the brain; moreover, abdominal titers were at the lower limit of detection (cycle threshold = 32–39), and we found no association between abdominal viral titer and mite level (N = 7 samples/mite level, Wilcoxon Test, Z = 0.13, P < 0.90). These results suggest that the degree of *Varroa* infestation in the current study generally did not immunocompromise the bees^25^. Moreover, mite infestation and pesticide susceptibility were uncorrelated (R^2^ = 0.0, P = 0.99), suggesting these are two independent outcomes of variation in early-life experience.

Retention of early-life social effects in an insect like the honey bee has been controversial because individuals undergo complete metamorphosis, i.e., restructuring of the body plan, during the transition to adulthood^26^. However, in the honey bee some brain progenitor cells are present during the larval stage and are retained through metamorphosis^27^. Thus brain molecular or structural variation in adults, perhaps as a result of epigenetic modifications during development, could retain social information across the pre-adult to adult transition^28^. Such epigenetic modifications could also occur in peripheral tissues. Here we have examined how the pre-adult social environment influences worker bees, and it is unknown whether such social effects may also occur in the other honey bee castes.

A number of different types of social cues could signal environmental variation to larvae. For example, colonies composed of more aggressive individuals may contain higher quantities of alarm pheromone (which is released as a volatile odor following aggressive arousal). Direct social interactions between larvae and ‘‘nurse’’ bees could also play a role: honey bee larvae are immobile and completely dependent on nurses who control the quantity and composition of larval food^29^. Moreover, more aggressive colonies may have better food resources because they tend to forage at higher rates^10^,^30^; lasting effects of larval pollen availability on adult behavior have been shown in other recent studies in the honey bee^31^. Nursing effects could even be non-nutritional; in rats, maternal licking and grooming during development influences stress response later in life^32^. The way in which variation in the larval environment influences aggressive behavioral output also is unknown: for example, larval environment could affect an individual’s motivation to respond aggressively to a non-nestmate intruder, but it could also influence the ability to detect nestmate cues and discriminate between nestmates and non-nestmates.

The molecular mechanisms underlying the broad behavioral and physiological effects of worker pre-adult environment are unknown; here we speculate about two possible candidate systems that have previously been implicated in both pesticide response and aggression in honey bees. First, variation in activity levels of cytochrome P450 detoxification enzymes is often associated with differential pesticide susceptibility^33^,^34^,^35^. Moreover, some of the same P450 enzyme classes involved in pesticide resistance show socially-responsive transcript abundance in the context of aggression in fruit flies^36^, and variation in transcript abundance as function of aggression in adult honey bees across several contexts (analyses of brains)^10^,^11^. In honey bees, P450 enzymes are modulated by dietary constituents in adult workers, and possibly larvae^37^,^38^. Thus perhaps larval diet has lasting effects on P450 levels in the brain and peripheral tissues, causing increased aggression and protection from pesticide toxicity during adulthood. P450 activity is directly involved in pesticide metabolism, but the link to behavior is less well understood^39^. It has been suggested that variation in P450 expression influences sensitivity to pheromones^36^, which could be applicable to the current results because odor perception is crucial to the aggressive response in the context of nest-mate recognition^13^.

In humans, early-life exposure to violence, which has similarly broad effects on behavior and physiology, is mediated at the molecular level at least in part by the evolutionarily conserved *NF-kappa B* signaling pathway, which is known to influence both neural plasticity and immune function in vertebrates^8^,^20^. In honey bees, studies in the brain have shown that *NF-kappa B* signaling pathways are significantly regulated following an aggressive encounter^7^, and models of transcription regulatory networks suggest *NF-kappa B* plays a central role in orchestrating the brain molecular changes that accompany variation in aggression^40^ (notably brain *NF-kappa B* mRNA itself is not differentially expressed as a function of variation in aggression^11^). Though work to-date cannot definitively address how *NF-kappa B* pathways influence neural signaling and thus behavior in the honey bee, one possibility is that variation in *NF-kappa B* pathway activity modulates cellular energy metabolism in the brain^41^; brain energy metabolic state has been causally linked to variation in aggression in the honey bee and fruit fly^12^,^42^.

Pesticide exposure in honey bees has been shown to inhibit *NF-kappa B* activity in whole-larvae^22^. If similar mechanisms operate in adults, it is possible that low-aggression bees (which show increased pesticide susceptibility) face a higher level of endogenous challenge to the innate immune system, and thus are more likely to die following immune-inhibiting pesticide treatment. Possible causes of immune challenge include pathogens such as fungal and viral infections, or the presence of pesticide residues within the colonies. Though we found no difference in DWV titers as a function of mite infestation in the current study^25^, we cannot rule out the possibility that other pathogens or pesticide residues that differed across high and low aggression colonies influenced immune function^43^,^44^,^45^.

High-aggression social environments may prime individuals to better withstand an immune challenge like pesticide treatment, e.g., by upregulating *NF-kappa B* or P450 activity in adult animals, similar to what occurs in humans in response to social stress. In humans however, immune activation in response to social stressors seems to lead to a harmful state of chronic inflammation^20^,^46^. Across animal species, aggression can be either positively or negatively associated with other fitness and health traits, presumably due to variation in the evolutionary drivers shaping aggression and associated phenotypes. The mechanistic basis of this variation, however, is largely unknown^47^. Elucidating the shared and unique mechanisms underlying the response to social aggression in animals should improve our understanding of resilience to adversity.

## Methods

### Host colony aggression assessment

The colony aggression assay was adapted from ref. 9. Briefly, we counted the number of bees that emerged from the colony entrance following a 60 s presentation of 5 μL of 1:10 isopentyl acetate (IPA) and mineral oil (Sigma Aldrich, St. Louis, MO, USA) applied to a piece of filter paper in the middle of the entrance. IPA alerts honeybees to a potential threat to the hive, and bees respond by crawling up to the edge and out of the entrance. More aggressive colonies show a greater response to IPA^9^. We quantified the response from photographs taken before and after IPA presentation.

### Treatment of cross-fostered individuals

One day prior to adult emergence, we removed brood frames from foster colonies, brushed off adult bees, and placed the frames in specialized “emergence boxes” in an incubator (34 °C). We collected emerging adult bees starting at 0800 the following morning. We transferred emerging bees from each frame into small petri dishes (100 × 20 mm, 8 bees/group in Trial 1, 6 bees/group in Trials 2 and 3) supplied with two 1.7 mL tubes filled with 40% sucrose. This method allowed us to easily standardize feeding treatments, and provided *ad libitum* sucrose for the duration of the experimental time period. In Trials 2 and 3 dishes were assigned a random number at the time of set-up so that behavioral assays were performed blind. All dishes were housed together in the 34 °C incubator and left undisturbed until behavioral assessment.

### Laboratory aggression assessment

When adult offspring were 8 days old, we moved dishes to a temperature controlled (25–30 °C) ventilated room under normal lighting conditions. We arranged dishes randomly and allowed 1 h for acclimation prior to testing. We assessed aggression using the Intruder Assay^12^. We collected intruders from colonies that were distinct from those used as egg sources or foster colonies because familiarity with colony odor affects aggressive response^48^. Behavioral scoring was adapted from ref. 12 and 49. Behaviors included aggressive antennation (antennation directed towards the posterior end of the intruder, sometimes accompanied by climbing on top of the intruder or following the intruder), antennation with mandibles open, biting, abdomen flexion (the abdomen is flexed but the stinger is not extruded), and stinging attempts (the stinger is visibly extruded). This entire range of behaviors is exhibited in response to colony intrusion by a non-nestmate or insect predator in a natural context (CCR, personal observation).

### Pesticide treatments

In Trial 2, we assessed cross-fostered bee mortality following treatment with acetamiprid. Following collections for behavioral analysis (see above), we collected 75 additional bees from each foster colony by source queen combination (N = 20) into large petri dishes (150 × 20 mm, provisioned *ad libitum* with 40% sucrose). When bees were 7 days old, we anesthetized the bees on ice and applied 1 μL of acetamiprid (dissolved in methanol) or methanol vehicle control to the thorax of each bee. Drug-treated and control-treated bees were housed separately in small (100 × 20 mm) petri dishes, provisioned *ad libitum* with 40% sucrose, placed back in the incubator, and assessed for mortality 24 h later.

### Mite data

We used mite sticky boards to estimate colony mite load^50^ for 25 colonies that were also surveyed for aggression (Fig. 1A, Pennsylvania). Mite counts for cross-fostered bees were tallied following behavioral assays for Trials 2 and 3. We counted all mites found on bees or in the petri dishes. Because there were no adult bees present at the time of offspring emergence, mites on cross-fostered bees most likely fed on the bees within the pupal cell during development^51^.

### Deformed wing virus assessment

At the time of emergence, we collected a subset of cross-fostered individuals for deformed wing virus (DWV) assessment. This was a distinct group of bees that had been collected and housed identically to the pesticide treated bees (see above). We anesthetized 7-day-old bees on wet ice and stored them at −80 °C for later viral analysis. For bees originating from 4 different genotype by foster colony combinations (we selected the two combinations with the highest mite loads and two with the lowest mite loads present on emerging bees), we pooled abdomens and brains separately into groups of 5 individuals (N = 3 or 4 groups of individuals from each frame, resulting in N = 7 pools per mite level, high and low).

We lyophilized heads and dissected brains on dry ice. We extracted RNA using the RNeasy Mini kit (Qiagen, Valencia, CA, USA). Brain homogenates were prepared by grinding tissue in 600 uL of lysis buffer with a hand-held tissue grinder, and RNA was extracted following manufacturer’s protocols. Abdominal homogenates were prepared using a FastPrep FP 120 Cell Disrupter (Qbiogene, Carlsbad, CA, USA): samples were removed from the −80 °C freezer and incubated on ice for 15 min. We then combined 700 μL of lysis buffer with 8, 0.7 mm zirconia beads (Biospec Products, Bartlesville, OK, USA) and performed two 45 s FastPrep cycles at top speed with a 10 min incubation on ice in between. We then centrifuged samples at 12,000 rcf for 30 s, pipetted 400 μL of lysate into a clean 1.7 mL tube, and proceeded with the extraction procedure following manufacturer’s protocols. We quantified total RNA using a Nanodrop spectrophotometer (Thermoscientific, Wilmington, DE, USA).

We synthesized cDNA from 200 ng RNA using ArrayScript (Ambion, Life Technologies, Grand Island, NY, USA) reverse transcriptase and a spiked-in internal control to confirm the quality of the synthesis. qRT-PCR was performed on an ABI Prism 7900 in triplicate 10 μL reactions in 384-well plates using PerfeCTa SYBR Green Fastmix (Quanta Biosystems, Gaithersburg, MD, USA). DWV titer was assessed using previously published primers and quantified against a cDNA standard curve generated from whole RNA for a honeybee showing viral symptoms (deformed wings). For abdominal samples, where DWV was detectable but at low levels (<1/2,000,000 of the concentration of the positive control), titers were normalized to the geometric mean of three constitutively expressed control genes: *eIF3-S8* (GB41874), *actin-1* (GB44311), and *gapdh* (GB50902). We then used a non-parametric test to compare normalized titers across groups.

### Statistical analysis

Statistical details are listed in the main text. All tests were performed using JMP 9.2.

## Additional Information

**How to cite this article**: Rittschof, C. C. *et al.* Early-life experience affects honey bee aggression and resilience to immune challenge. *Sci. Rep.* **5**, 15572; doi: 10.1038/srep15572 (2015).

## Supplementary Material

Supplementary Information
