# Supplementary material for: Early-life experience affects honey bee aggression and resilience to immune challenge
Source: Sci Rep. 2015 Oct 23;5:15572. doi: 10.1038/srep15572 (PMC4616062; doi:10.1038/srep15572)
Supplement: Supplementary Information [file srep15572-s1.pdf]

SUPPLEMENTARY INFORMATION

Early-life experience affects honey bee aggression and resilience to immune challenge

Clare C. Rittschof, Chelsey B. Coombs, Maryann Frazier, Christina M. Grozinger, Gene E. Robinson

Supplementary Figures

a

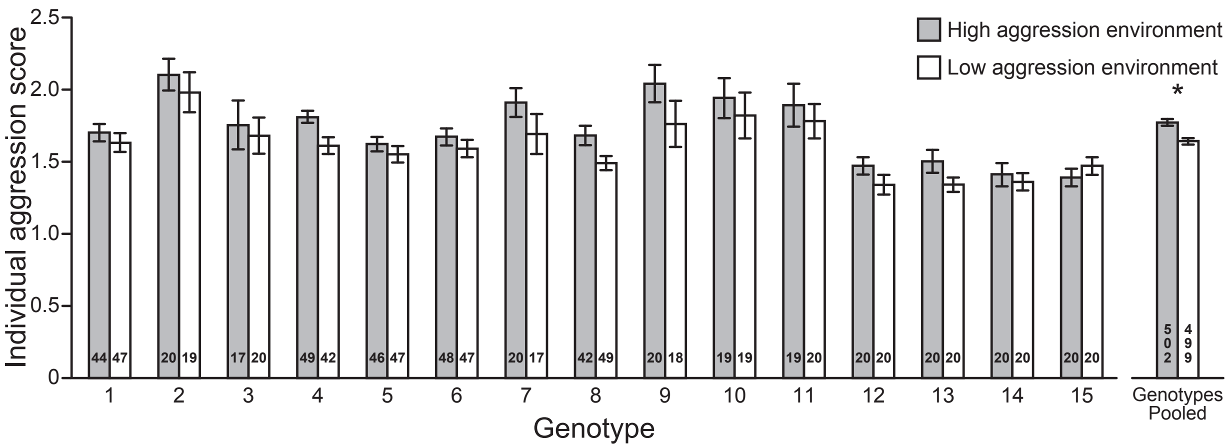

b

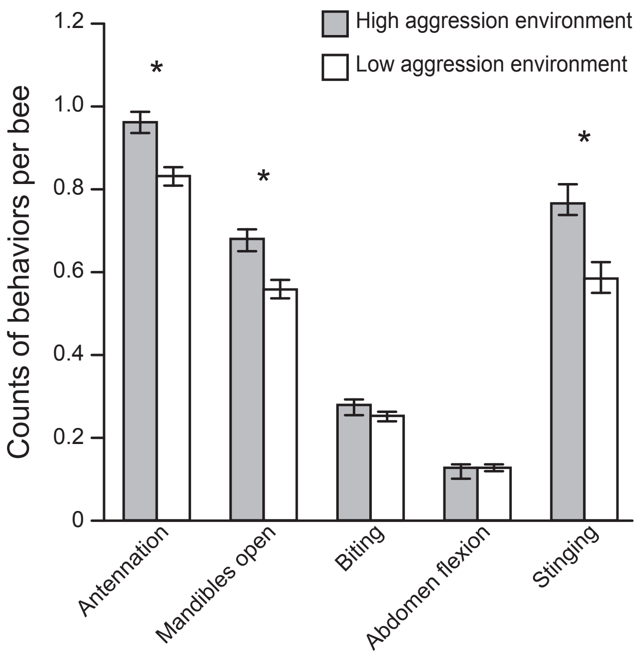

Figure S1. a. The effects of pre-adult social environment on behavior were consistent across genotypes. In a nested analysis of pooled z-scores across all three trials, we assessed environmental effects for the 15 genotypes for which siblings were raised in both a high and a low aggression colony in the same trial. Sample sizes are denoted in the bars, and are variable due to some mortality prior to behavioral assessment. Sample sizes are doubled for genotypes assessed in Trial 3 (1,4,5,6, and 8) because all genotypes were housed in two high and two low aggression foster colonies. In 14 out of 15 cases, siblings raised in high aggression colonies were more aggressive as adults relative to siblings kept in low aggression colonies (data shown are mean +/- S.E.M. of log-transformed raw scores, nested analysis of z-scores: foster colony aggression [genotype]: $F_{15} = 1.55$, $P<0.08$). An analysis of all data pooled across three trials (48 total genotype by foster colony combinations, sample sizes denoted in bars) showed highly significant effects of environment and a moderate effect of genotype on aggression (data shown are mean +/- S.E.M. of log-transformed raw scores, significance for colonies pooled indicated by asterisk; analysis of z-scores: whole model: $F_{18,982}=2.43$, $P<0.0008$, foster colony aggression level: $F_1=17.3$, $P<0.0001$, worker genotype: $F_{17}=1.6$, $P<0.069$ (the interaction term was non-significant and so excluded from the final model). **b.** Social effects were observed across a range of aggressive behaviors. Colony environment had significant effects on both high and low level aggressive behaviors (mean +/- S.E.M., two-tailed *t*-test $P<0.05$ is indicated by asterisks, $N_{High\ aggression}=502$ groups, $N_{Low\ aggression}=499$ groups). Data are represented as counts averaged across the number of bees in the group.

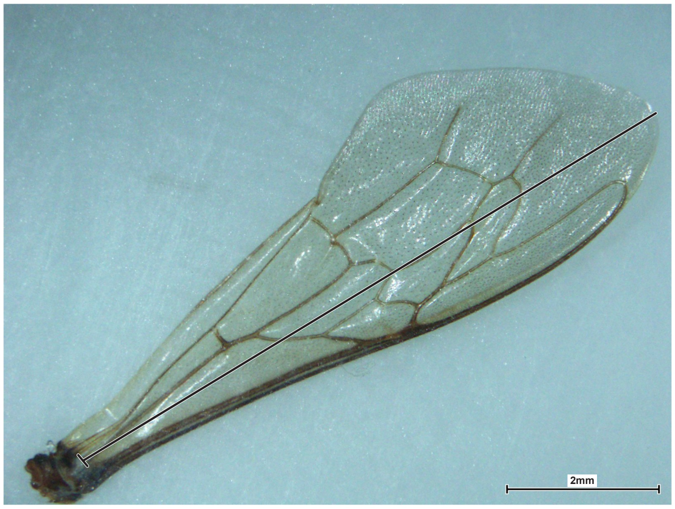

Figure S2. Sample image of wing measurements used for body size. The black line spanning from the wing base to the wing margin represents the length measurement assessed.

a

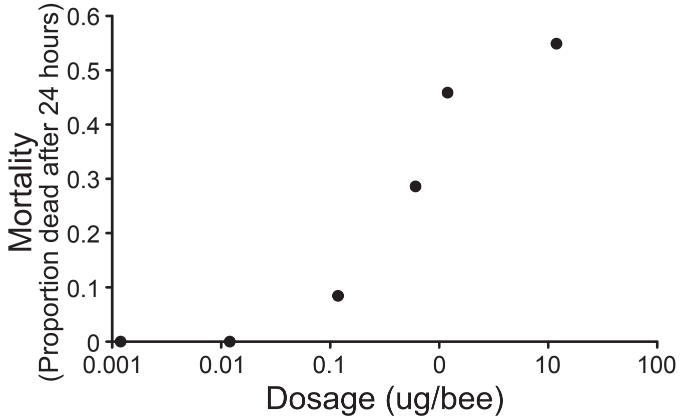

b

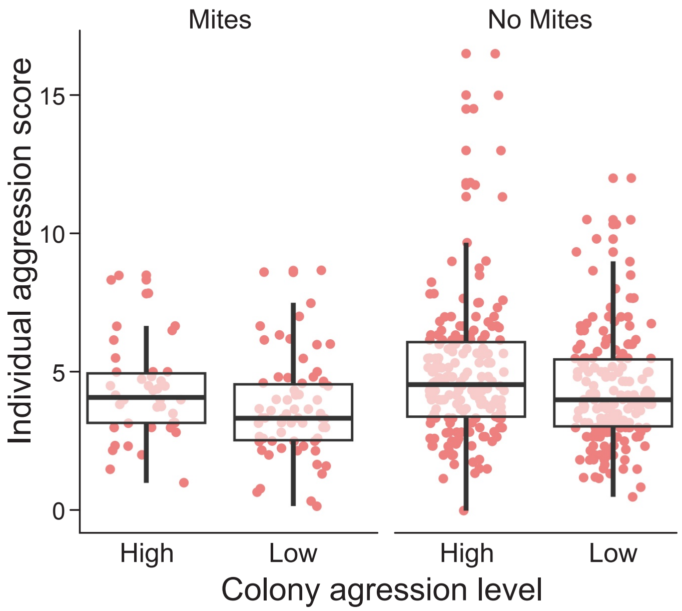

Figure S3. a. Dose-response curve for acetamiprid following a 1 µL topical application to the thorax of 7-day-old lab reared bees from a single genotype (N=20 bees/dose). The relationship between mortality and dosage appears to be non-linear, but our selected dose, 1.2 µg/bee, is an approximate LD_{50}. Dosages assessed were based in part on information available from the EPA Ecotoxicity Database. Bees assessed here were housed and fed similarly to bees in the main experiment. The relatively low mortality rates in Trial 2 (0-36%) compared to this pilot dose-response curve could reflect natural variation in susceptibility as a function of genotype or developmental environment. **b.** Individual aggression scores as a function of mite presence or absence in Trial 3. Aggression score was significantly influenced by both foster colony aggression level and the presence of mites on emerging offspring (whole model: $F_{3,456} = 6.44$, $P<0.0003$; foster colony aggression: $F_1=7.29$, $P<0.007$, mite presence: $F_1=7.30$, $P<0.008$, interaction: $P<0.7$). Data shown are raw scores, and dots represent individual groups assayed for behavior. Boxplots show medians, 1st and 3rd quartiles, and interquartile range. Colony

aggression level influenced individual aggression scores similarly regardless of mite presence, suggesting mite presence alone does not adequately explain the major social influences we observed.

Supplementary Methods

We performed three separate trials for the cross-fostering experiment. Trial 1 took place during August 2013 in Urbana, Illinois. Trials 2 and 3 took placed during June and July 2014 in State College, Pennsylvania. Trials are detailed below.

Bee sources

To choose host colonies, we sampled aggression for a total of 38 colonies in Illinois (IL, 2013) and Pennsylvania (PA, 2014) (Fig 1a). Aggressive response ranged from -16 to 244 bees (mean = 29 +/- S.D. = 52.3), indicating a broad range of natural variation, including some colonies that retreated in response to a threat[1]. PA colonies showed higher variation in aggression compared to IL colonies, likely due to differences in management practices or genetic background.

The 12 IL colonies represented a mixture of European honeybee subspecies, primarily *Apis mellifera* ligustica. Colonies had variable management histories prior to the start of the study (including occasional brood removal), though colonies were left undisturbed following cross-fostered brood introduction. The 26 PA colonies were established in April 2013 from nucleus colonies and packages purchased from Gardner Apiaries (Baxley, GA, USA) and Bob Brochman (Russian, WV, USA). Colonies represent contributions of queen stocks from Miska, FL, Weaver, TX, Palmer, VT, and Russian, WV. From establishment until use in the current studies, PA colonies were left mostly undisturbed (no brood or honey removed).

Host colony aggression assessment and selection details

We performed aggression assessments early in the morning (0730-0930 depending on ambient temperature) when there was little foraging traffic at the colony entrance. Colonies were sampled serially (10-15 s apart) in random order. Because assays were performed quickly and colonies were sufficiently spaced within each apiary, it is unlikely that cues drifted among colonies during the assays.

For Trial 1, we sampled 12 colonies across three apiaries and selected 8 colonies (two in each of two apiaries, four in one apiary). For Trial 2, we sampled 26 colonies across three different apiaries. We selected 10 colonies from 3 different apiaries (two in each of two apiaries, and 6 in the third apiary). For the 10 foster colonies in Pennsylvania, we resampled aggression 3 days following the first measurement to confirm colony aggression was consistent and repeatable ($R^2 = 0.5$, $P<0.022$). In Trial 3 we selected four colonies (two high and two low aggression across two apiaries) that were used in Trial 2.

For the 18 colonies used to host brood, we counted hive boxes as a non-invasive estimate of colony size, but found no relationship between size and aggression ($N=18$, $R^2 = 0.013$, $P<0.65$). These results are consistent with previous studies showing that honey bee aggression is highly variable, even among similar-sized colonies, and stable over time[2].

Host colony mite data

Colony mite data was collected on June 17, 2014. For the regression analysis presented in Fig 2b, we omitted two outliers, one high and one low aggression colony, with mite numbers greater than 3 standard deviations above the global mean.

Cross-fostered individual source genotypes and experimental designs

In order to evaluate behavioral effects of the pre-adult environment across a range of genotypes, we fostered source eggs from 18 unique queens across the three trials (4 queens in Trial 1, 10 in Trial 2, 4 in Trial 3 plus one queen which was reused from Trial 2). Naturally mated queens (mated with ~12 drones on average) were selected randomly from colonies that were not used to foster brood. To collect eggs, we used specially designed cages to restrict queens to an empty honeycomb frame within their home colonies for 12 h (Trial 1) or 24 h (Trials 2 and 3). We then removed the frame, inspected the frame for eggs, marked the location of the eggs, and transferred frames of eggs to the brood nest of host colonies within 2 h. In order to sample a single genotype across more than one colony environment, we collected eggs over multiple days: 2 consecutive days (Trials 1 and 2), and 4 days (Trial 3; two sets of 2 consecutive days with a day in between).

In Trial 1, host colonies were paired randomly *a priori* by apiary and aggression level (one high aggression paired with one low aggression), and the same genotypes were introduced into both colonies of each pair (4 genotypes across 8 colonies). In Trial 2 we used 10 genotypes and 10 different host colonies. To increase sampling, we introduced eggs from each of the 10 genotypes into two unique colonies (resulting in 20 unique genotype by colony combinations). In order to account for possible effects of date on behavior, we randomized the day of introduction and the assignment of genotype to host colony. To do this, we randomly assigned the 10 host colonies to two "set-up" days (5 colonies were set up each day). We distributed eggs from the 10 genotypes to 5 colonies on day 1 (2 genotypes randomly assigned to each colony), and repeated this procedure on set-up day 2. As a result of this randomized design, some genotypes were introduced into two high or two low aggression colonies. However 6 of 10 genotypes were sampled across both a high and low aggression environment in Trial 2. In Trial 3, we collected eggs laid by 5 queens over four days, and we introduced one set of five genotypes to each of 4 host colonies, selecting the order of introduction at random. We introduced all five frames to a single colony at once to minimize disturbance to the foster colony. Despite the differences in experimental design, our behavioral results were consistent across all three trials (Fig 1b).

Laboratory aggression assay

Intruders were vacuumed from the colony entrance and kept in plexiglass boxes with 40% sucrose until use in the experiment. Feeding minimizes variation in intruder behavior. There were differences across the three trials in how stinging activity was quantified during the aggression assay, leading to differences in overall aggression scores (e.g., Fig 1b). In Trials 1 and 3, we recorded an instance of a sting attempt a single time, regardless of the amount of time the bee spent stinging. In Trial 2, we tallied the occurrence of the attempt, and marked an additional tally every 10 s an individual continued to sting. This method kept track of the duration of the attempt, which is a measure of intensity, but generally resulted in higher aggression scores for that trial. Despite differences in scoring method, we observed consistent treatment effects across all three trials. Tallies of each behavior were multiplied by a severity index (1-5) and then

summed to arrive at the total aggression score[3]. Notably, both high and low level aggressive behaviors varied as a function of colony environment, suggesting a robust effect (Fig S1b).

Body size measurements

We assessed the relationship between host colony aggression and offspring body size for 3 of the 4 genotypes used in Trial 1 (N=20 bees per queen and colony combination). We did not assess body condition because some brood frames contained honey at the time they were removed from the colonies and placed in the incubator, and thus it was difficult to control food availability within the first few hours of emergence. For each bee, we took a digital picture of one forewing. We analyzed images using the "measure" tool in ImageJ v1.47 (Wayne Rasband, National Institutes of Health, USA). To standardize our measurement procedures, we first drew a line across the widest part of the median plate at the wing base[4]. We then drew a second line perpendicular to the first beginning from the first line to the most distal point of the wing. This line always crossed the second radio-medial cross-vein (third submarginal cross-vein) described by Michener[5]. We measured this second line and used a scale bar imbedded in each image to calculate wing length (mm).

Cross-fostered bee mite assessment

In Trials 2 and 3, overall offspring mite numbers were low, with a median of 0 mites per group for both trials. In Trial 2, 27% of groups had mites (0-8 mites/group), and in Trial 3, 22% of groups had mites (0-4 mites/group). For the 10 unique host colonies, field and offspring mite counts were highly correlated ($R^2=0.92$, $P<0.0002$) with two exceptions, including a high aggression colony with lower than predicted offspring mite numbers, and a low aggression colony showing the opposite pattern. Thus it is possible that high aggression colonies are more efficient at removing mites, minimizing the number of mites present on emerging offspring.

Supplementary References

1. Kastberger, G., Thenius, R., Stabentheiner, A., and Hepburn, R. (2008). Aggressive and Docile Colony Defence Patterns in Apis mellifera. A Retreater–Releaser Concept. J. Insect. Behav. *22*, 65-85.
2. Wray, M.K., Mattila, H.R., and Seeley, T.D. (2011). Collective personalities in honeybee colonies are linked to colony fitness. Animal Behaviour *81*, 559-568.
3. Richard, F.J., Holt, H.L., and Grozinger, C.M. (2012). Effects of immunostimulation on social behavior, chemical communication and genome-wide gene expression in honey bee workers (*Apis mellifera*). BMC Genomics *13*, 558.
4. Snodgrass, R.E. (1910). The anatomy of the honey bee, (Washington, D.C.: Government Printing Office).
5. Michener, C.D. (2007). The bees of the world, Second Edition, (Baltimore, MD: The Johns Hopkins University Press).
